# Neural representations of the content and production of human vocalization

**DOI:** 10.1073/pnas.2219310120

**Published:** 2023-05-30

**Authors:** Vera A. Voigtlaender, Florian Sandhaeger, David J. Hawellek, Steffen R. Hage, Markus Siegel

**Affiliations:** ^a^Department of Neural Dynamics and Magnetoencephalography, Hertie Institute for Clinical Brain Research, University of Tübingen, 72076 Tübingen, Germany; ^b^Centre for Integrative Neuroscience, University of Tübingen, 72076 Tübingen, Germany; ^c^Magnetoencephalography (MEG) Center, University of Tübingen, 72076 Tübingen, Germany; ^d^Graduate Training Centre of Neuroscience, International Max Planck Research School, University of Tübingen, 72076 Tübingen, Germany; ^e^F. Hoffmann-La Roche, Pharmaceutical Research and Early Development, Roche Innovation Center Basel, 4051 Basel, Switzerland; ^f^Neurobiology of Social Communication, Department of Otolaryngology - Head and Neck Surgery, Hearing Research Centre, University of Tübingen, 72076 Tübingen, Germany

**Keywords:** human speech, vocalization, MEG, MVPA, neural information

## Abstract

Speech, as the spoken form of language, is fundamental for human communication. The phenomenon of covert inner speech implies functional independence of speech content and motor production. However, it remains unclear how a flexible mapping between speech content and production is achieved on the neural level. To address this, we recorded magnetoencephalography in humans performing a rule-based vocalization task. On each trial, vocalization content (one of two vowels) and production form (overt or covert) were instructed independently. Using multivariate pattern analysis, we found robust neural information about vocalization content and production, mostly originating from speech areas of the left hemisphere. Production signals dynamically transformed upon presentation of the content cue, whereas content signals remained largely stable throughout the trial. In sum, our results show dissociable neural representations of vocalization content and production in the human brain and provide insights into the neural dynamics underlying human vocalization.

## Significance

Speech research is largely bound to the study of humans and, thus, suffers from methodological limitations. Therefore, key mechanisms of speech production remain unclear. One central question is whether neural representations of speech content and motor production can be dissociated. We addressed this with an innovative paradigm where we combined magnetoencephalography, advanced multivariate pattern analysis, and a rule-based vocalization task. We could dissociate neural representations of content and production and further describe their temporal and spatial dynamics. Our results suggest that content has an abstract representation that allows to generalize across different production forms. With this study, we answer some essential questions of speech research and provide a fruitful framework for further noninvasive research.

Vocal behavior is an essential component of human communication. Particularly speech, the spoken form of language, is a highly sophisticated skill exclusive to humans. Thereby, we can encode information not only in sound (overt speech) but also in thought (covert speech). These different speech forms imply functional independence of speech content and motor production. However, it remains an open question how content and production are represented neuronally and how the brain achieves a flexible mapping between the two.

In speech, two levels need to be distinguished. The lexical level, which refers to entire words, and the sublexical level, which refers to parts of words, such as phonemes (single vowel or consonant) or syllables (more than one vowel or consonant) (1). On both levels, speech engages a broad cortical network comprising the primary motor cortex (M1), premotor and supplementary motor cortices (PMC and SMA), as well as sensory and auditory cortices (2–8). Compared to phonemes, more complex sublexical speech leads to stronger activation in parts of the network (8), whereas lexical speech recruits additional areas for high-level speech processes like word selection and combination (6, 9, 10).

Several findings suggest that there is a neural representation of speech content which is to some degree independent of its motor production. This independence is intuitive on the lexical level. Indeed, on the lexical level, overt and covert speech were found to share a common cerebral network with similar activation patterns (11–17), which differ primarily in activation magnitudes due to a different degree of executive motor control (15, 18). Furthermore, Broca’s area was found to act as a supramodal hub, exhibiting language-specific activation independent of the production form (19).

The motor-independent representation of speech content is less intuitive on the sublexical level, where content may be expected to be more tightly bound to motor production. Still, initial evidence also suggests production-independent representations of sublexical entities like syllables and phonemes. Efference copies in overt and covert speech were found to have similar patterns not only on the lexical but also on the sublexical syllable level, except for M1 recruitment exclusive to overt speech (20–23). Furthermore, specifically on the syllable level, covert speech was found to yield articulatory representations in premotor regions, as well as acoustic representations in sensory and auditory cortices similar to overt speech (24). On the phoneme level, evidence remains sparse as most studies have been related to motor production (25–28) and efference copies were so far only described in overt speech (29). Yet, indirect evidence from phoneme-related speech errors in covert speech suggests a motor-independent phoneme representation (30).

In sum, past work suggests that neural activity underlying lexical and sublexical vocalizations represents both speech content and motor production. However, the representational overlap of content and motor representations and to what extent it is possible to dissociate these aspects remain unclear. Furthermore, the dynamic interplay between emerging representations of content and motor production is not known, as most previous studies used neural data with inherently poor temporal resolution. These questions are particularly open on the phoneme level, where direct neuronal evidence is missing.

To address these questions, here we aimed, first, to independently manipulate and decode neuronal content and motor components of human phoneme vocalization and, second, to investigate their dynamic interplay across time. We recorded magnetoencephalography (MEG) while subjects performed a rule-based vocalization task dissociating the content and motor aspects of sublexical speech. Content (one of two vowels) and production (overt or covert) were instructed sequentially and in random order. Multivariate pattern analysis (MVPA) of time-resolved MEG data allowed us to characterize the format, overlap, and temporal dynamics of neural content and production representations.

With this approach, we were able to read out content and motor information several seconds before speech onset. The strength of neural information correlated with the degree of motor involvement. At the beginning of the trial, when only one variable was known, the isolated representations of content and production were similar. The production representation transformed once the content was known, whereas the content representation remained stable until the onset of vocalization.

## Results

### The Components of Vocalization Can Be Decoded Independently.

**Fig. 1.**
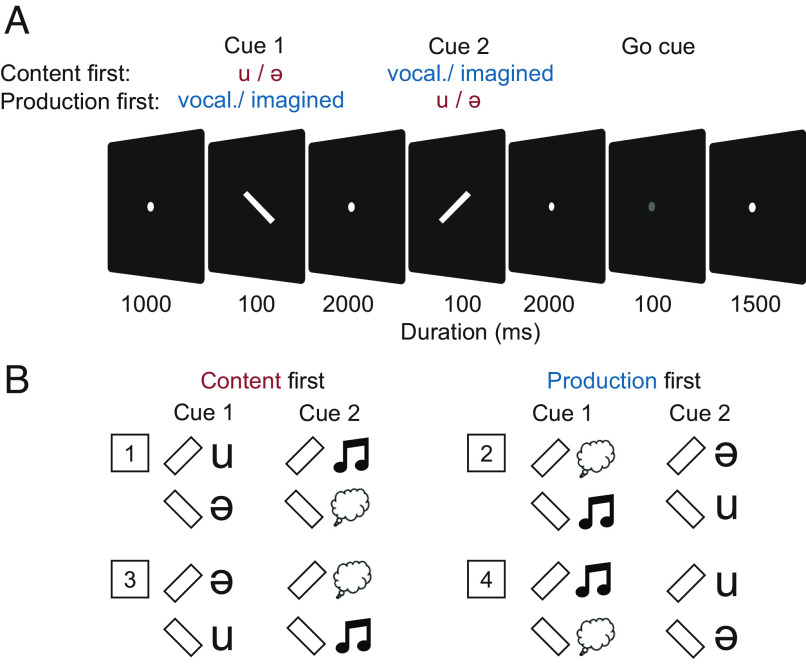
Rule-based vocalization task. Participants imagined or vocalized different contents (phonemes /u/ and /ə/). (*A*) Production and the content were instructed successively with visual cues, according to the respective rule of the trial block. (*B*) Rules for four trial blocks per recording session. In two blocks, the content was instructed first; in the other two blocks, the production was instructed first.

We recorded MEG while subjects performed a rule-based vocalization task. Participants had to overtly vocalize or covertly imagine the vocalization of two different vowels. During each trial, content (/u/ / /ə/) and production (vocalized/imagined) were instructed sequentially with visual cues (Fig. 1*A*). Each cue lasted 100 ms and was followed by a 2-s delay. At the end of the trial, a brief dimming of the fixation point served as a go cue for the onset of vocalization or imagination. The order of instruction was randomized, as was the assignment of the instructed content or production to the visual cues (Fig. 1*B*). Participants performed the correct production type (vocalized vs. imagined) in 97.98% of the trials. In case of vocalized trials, the correct vowel (/u/ vs. /ə/) was performed in 100% of the trials. We checked vocalized trials for their onset latency (possible for 37 sessions). In 98.48% of the trials, the vocal onset was after the go cue, as instructed. The mean vocal latency of these trials was 0.58 s (±0.12 SD).

For each subject and recording session, we computed neural information about content and production. We applied cross-validated multivariate analysis of variance (cvMANOVA) on preprocessed single-trial MEG data from all sensors (see *Methods*; see *SI Appendix*, Fig. S1 for example single-trial data) (31, 32). The resulting measure of neural information can be interpreted analogously to classifier performance from multivariate decoding analyses. To enable robust statistical tests, we averaged information in the time windows between cues 1 and 2 (delay 1), as well as between cue 2 and the go cue (delay 2).

**Fig. 2.**
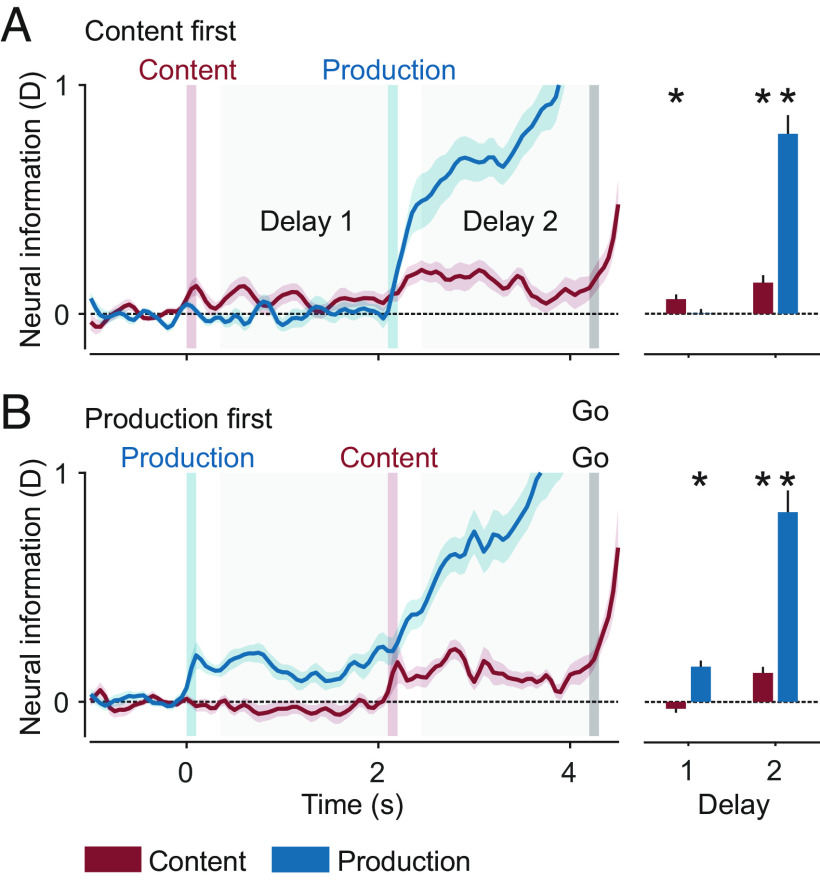
Neural information about content and production. (*A*) Information in trials with content instructed first. (*B*) Information in trials with production instructed first. Shaded regions and error bars indicate SEM. Bar plots show average information in delays 1 and 2. Asterisks indicate significance (n = 24, *P* < 0.05 corrected; *t* test, one tailed).

We observed significant neural information about both variables (Fig. 2 and *SI Appendix*, Fig. S2 with individual data points). For both orders of cue presentation, we found information about the variables shortly after their respective instruction. When content was instructed first, there was significant content information in delay 1 (*P* = 0.003; corrected) and significant information about content and production in delay 2 (content: *P* = 3.4 × 10^−4^, production: *P* = 1.6 × 10^−9^; corrected). Conversely, when production was instructed first, content information was only present in delay 2, whereas production information was highly significant in both delays (content: p_del2_ = 7.8 × 10^−5^; production: p_del1_ = 2.8 × 10^−6^, p_del2_ = 8.5 × 10^−9^; corrected). Thus, both the content of a vocalization and its production form were represented neuronally, several seconds before the actual execution.

### The Components of Vocalization Are Modulated by Effort.

**Fig. 3.**
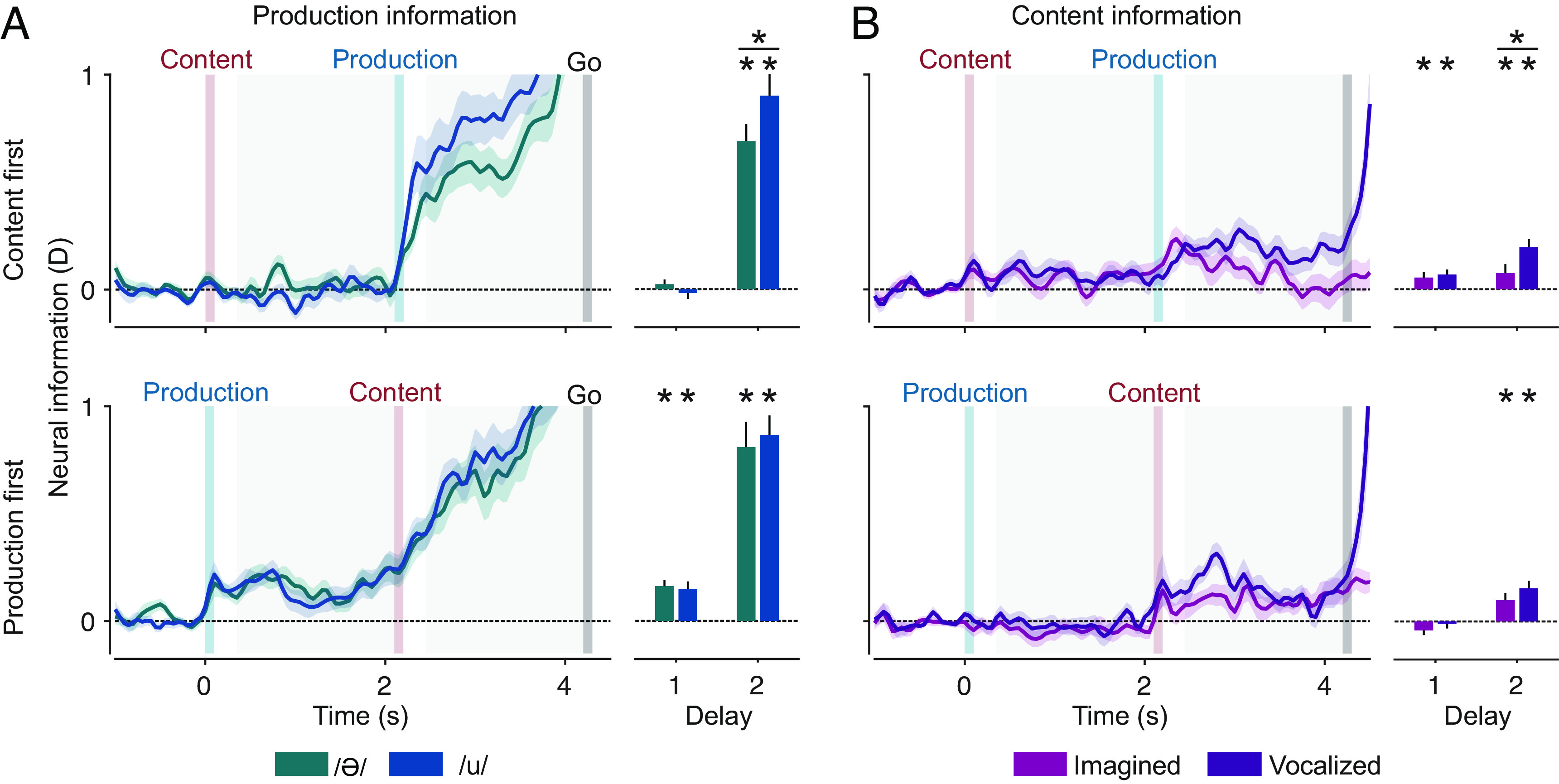
Neural information about content and production in split conditions. (*A*) Production information in both orders. (*B*) Content information in both orders. Shaded regions and error bars indicate SEM. Bar plots show averaged information of delays 1 and 2. Asterisks above individual bars indicate significant information (n = 24, *P* < 0.05 corrected; *t* test, one tailed). Horizontal lines with asterisks on top indicate a significant difference (n = 24, *P* < 0.05 corrected; paired *t* test, two tailed).

Both experimental dimensions entailed differences in motor effort. Imagined vowels lacked actual vocalization, just as /ə/, as a nonarticulated vowel, lacked the strong articulation of /u/. Therefore, we wanted to investigate whether the neural information about content was equally strong for both production types and whether the neural information about production type was identical for both vowels. To test this, we decoded content separately for both production types and production separately for both vowels (Fig. 3 and *SI Appendix*, Fig. S3 with individual data points).

In all conditions, information about both variables could be decoded after their respective instruction. When content was instructed first, production information was significant for both vowels, in delay 2 (/ə/: *P* = 7.8 × 10^−9^, /u/: *P* = 6 × 10^−9^; corrected). When production was instructed first, it could be read out in both delays and for both vowels (/ə/: p_del1_ = 4.1 × 10^−6^, p_del2_ = 4.8 × 10^−7^; /u/: p_del1_ = 1.4 × 10^−4^, p_del2_ = 1.6 × 10^−9^; corrected). In the second delay, production information was higher in /u/ than in /ə/ trials, but only significantly so when content was instructed first (*P* = 0.026, paired *t* test; corrected).

Content information was significant in both delays and production types when content was instructed first (imagined: p_del1_ = 0.044, p_del2_ = 0.044; vocalized: p_del1_ = 0.004, p_del2_ = 2.7 × 10^−5^; corrected). When production was instructed first, content information was only significant in the second delay, again in both production types (imagined: *P* = 0.007, vocalized: *P* = 1.5 × 10^−4^; corrected). Content information was higher during vocalized than imagined trials, in both orders and all relevant delays. This difference was significant in the second delay when content was instructed first (*P* = 0.007, paired *t* test; corrected).

In sum, both production and content information were present in all individual conditions and, furthermore, higher in those conditions with stronger motor involvement.

### The Components of Vocalization Are Represented in Cortical Speech Areas.

**Fig. 4.**
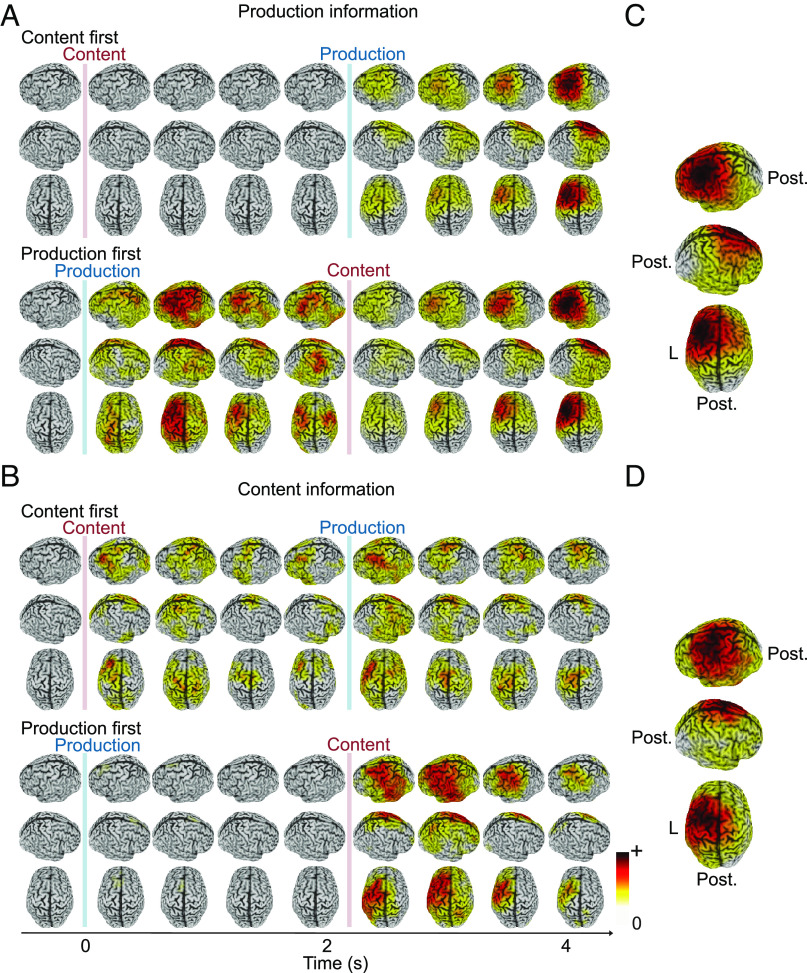
Spatial dynamics of neural information about content production. (*A*) Production information in both orders. (*B*) Content information in both orders. Left, right, and top views of the cortical distribution of information. Information was averaged in 500-ms intervals, except for the intervals directly after the cues, for which the first 250 ms was excluded. (*C* and *D*) Averaged information over all three delays where information was available for the respective variable. Information strength is color coded, as indicated by the color bar (white: zero or very low information, red: high information).

We found that vocalization content and production were represented in cortical areas typically associated with speech. To characterize the cortical distribution of content and production information, we repeated the cvMANOVA analysis on the source level using a searchlight approach. We then averaged neural information within four 500 ms time windows per delay. This analysis revealed spatially stable representations of both variables (Fig. 4) with similar and broad frontocentral distributions that included M1, SMA, and Broca’s area (see *SI Appendix*, Fig. S4 for information in all areas according to the automated anatomical labeling (AAL) atlas).

**Fig. 5.**
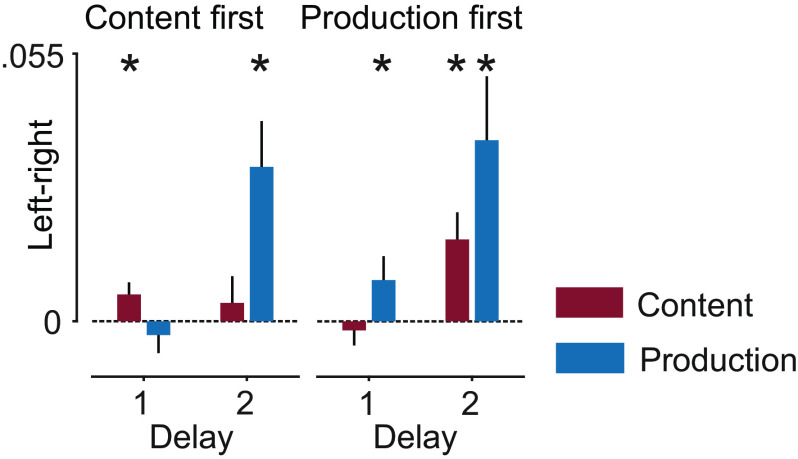
LI of content and production information. The LI (left – right hemisphere information) was computed for both delays and both orders. Asterisks indicate significance (n = 24, *P* < 0.05 corrected; *t* test, one tailed).

For higher-order language processes, neural activity is known to be left-lateralized, which is debated for speech on the sublexical level (28, 33). Our source patterns suggested a clear left lateralization. We tested this by computing a lateralization index (LI) for both variables (Fig. 5 and *SI Appendix*, Fig. S5 with individual data points). Both variables were left-lateralized in all delays after the respective instruction. This was significant for content information in the first delay when it was instructed first (*P* = 0.037; corrected) and in the second delay when production was instructed first (*P* = 0.006; corrected). Production information was significantly left-lateralized in both delays when production was instructed first (p_del1_ = 0.048, p_del2_ = 0.009; corrected) and in the second delay when it was instructed second (*P* = 0.003; corrected).

Taken together, source analysis showed that both content and production had stable left-lateralized representations on the cortical level. This did not only identify the origin of neural information within well-known speech-associated areas but also excluded confounds due to inherently nonlateralized effects of visual cues or electromagnetic artifacts.

### The Components of Vocalization Have Different Representational Formats.

**Fig. 6.**
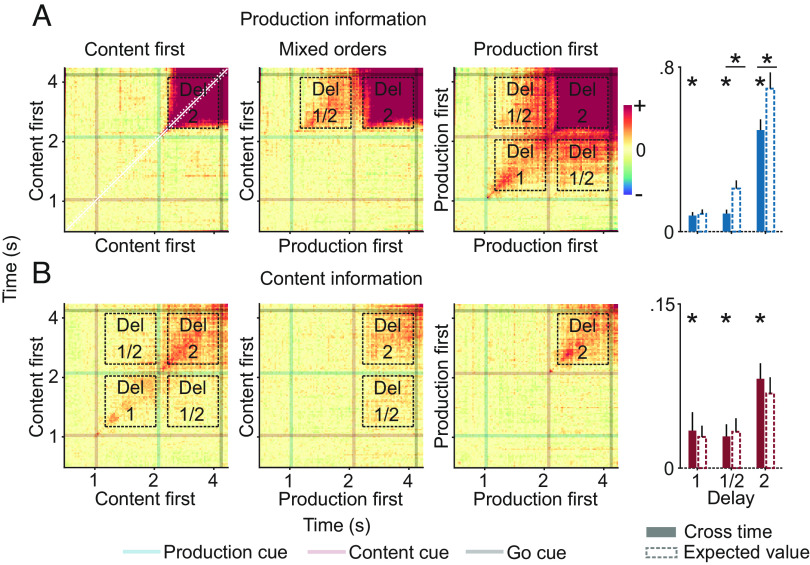
Cross-time information about content and production. (*A*) Cross-time production information. (*B*) Cross-time content information. Bar plots show the averaged cross-temporal information for delays 1, 2, and 1/2, as well as the expected values for stable representations. The diagonals ± 100 ms were excluded from calculations. Dashed squares indicate the averaged time windows. Asterisks indicate significant information (n = 24, *P* &lt; 0.05 corrected; *t* test, one tailed), and horizontal bars with asterisks on top indicate significantly smaller cross-information than the expected value (n = 24, *P* &lt; 0.05 corrected; paired *t* test, one tailed).

The searchlight analysis indicated spatial stability of the coarse cortical distribution of content and production information across time. Nonetheless, the representational format, i.e., the fine-grained cortical pattern underlying each representation, may be dynamic. To test whether neural representations of content and production transformed across time, we cross-decoded both variables on the sensor level across time (34) (Fig. 6). Stable or dynamic representations would yield high or low cross-time decoding, respectively. We performed this analysis both on the content-first condition and on the production-first condition, as well as training on all timepoints of one condition and testing on those of the other (Fig. 6, mixed orders).

For statistical testing, we averaged neural cross-information per variable and delay such that neural information about content or production was in principle accessible at all training and test timepoints included in the statistical analysis (Fig. 6, dashed squares and bar plots). Within both delays 1 and 2, there was significant cross-temporal information about both variables (production: p_del1_ = 6.3 × 10^−5^, p_del2_ = 2.9 × 10^−9^; content: p_del1_ = 0.027, p_del2_ = 1.1 × 10^−5^; corrected). There was also significant cross-temporal information between delays 1 and 2 for both variables (production: *P* = 6.3 × 10^−5^; content: *P* = 0.012; corrected).

To test whether representations significantly differed between time points, we computed an estimate of the expected cross-time-information in case of perfectly stable representational formats but potentially different information magnitudes (32). The cross-temporal stability of production representations was lower than expected in all time windows and significantly so between delay 1 and 2 and within delay 2 (p_del1/2_ = 2 × 10^−4^, p_del2_ = 7.3 × 10^−6^; corrected). In contrast, cross-temporal content information was never significantly smaller than expected for a temporally stable representation. Thus, while we found evidence for a partially dynamic representation of production type, this was not the case for content, which appeared stable over time.

**Fig. 7.**
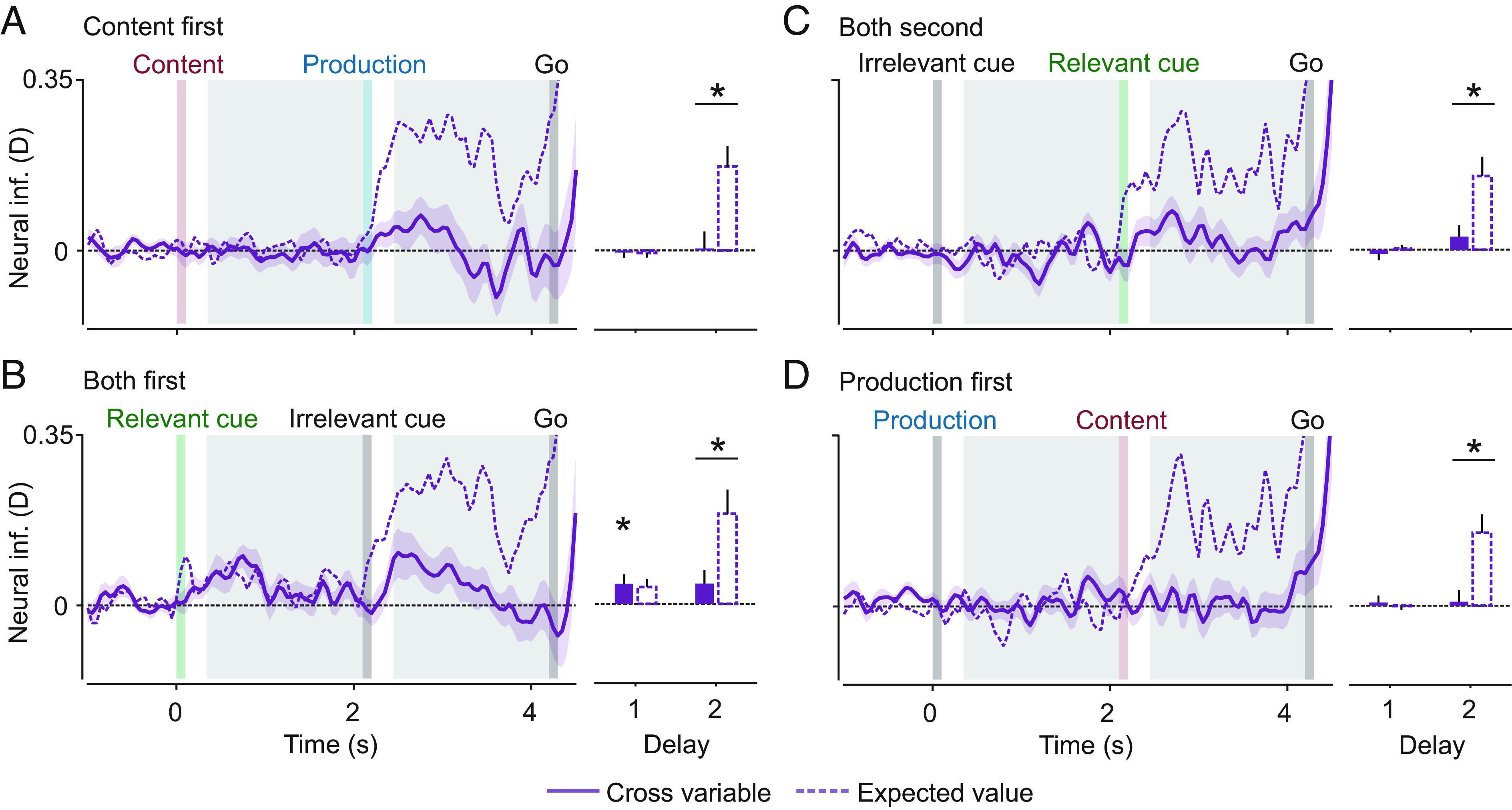
Cross-variable information of content and production. Cross-information in both orders and mixed orders with the relevant cue first or second. (*A*) Cross-information in trials with content instructed first. (*B*) Cross-information across trials with relevant cue first. (*C*) Cross-information across trials with relevant cue second. (*D*) Cross-information in trials with production instructed first. Solid lines indicate cross-information, and dashed lines indicate the respective expected values for identical representations. Bar plots show the averaged cross-information and the averaged expected values. Asterisks indicate significant cross-information (n = 24, *P* < 0.05 corrected; *t* test, one tailed), and horizontal bars with asterisks on top indicate significantly smaller cross-information than the expected value (n = 24, *P* < 0.05 corrected; paired *t* test, one tailed).

To what extent are the neural representations of content and production similar? Our cross-time decoding analysis showed that both representations evolve differently across time. We took this as an indication that these representations are not identical. To rigorously estimate the extent of representational overlap, we implemented a cross-variable analysis, training the algorithm on one variable and testing it on the other. We computed cross-variable information in both cue orders and in mixed cue orders where the relevant cue was either first or second. Again, we compared the observed cross-information to the cross-information expected under the assumption of identical representations (Fig. 7).

We found significant cross-information only in delay 1 in the order with the relevant cue first (*P* = 0.046; corrected). In the other orders, as information about both variables could only be present after the second cue, no cross-information was expected in delay 1. While we observed a small amount of cross-information in delay 2 of the mixed orders, this was not significant. On the other hand, cross-information was significantly smaller than its expected value in the second delay of all orders (content first: *P* = 0.004; both first: *P* = 0.02; both second: *P* = 0.01; production first: *P* = 0.002; corrected). Taken together, these results show that content and production representations overlap but are not identical. In our data, isolated content representations (before knowledge of the production type) cannot be distinguished from isolated production representations (before knowledge of the content). Thus, content and production representations are indistinguishable as long as the respective other variable is unknown to the participant. However, as soon as both aspects become available, their representations show strong differences.

### Information about the Components of Vocalization Remains Stable across Sessions.

**Fig. 8.**
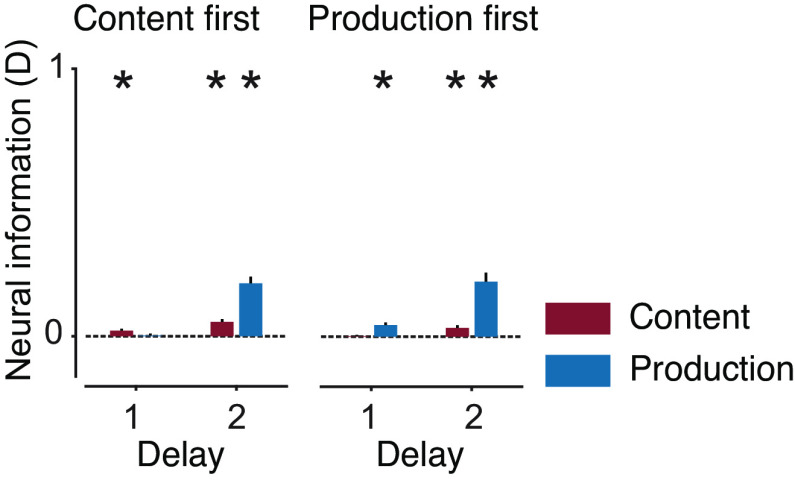
Cross-session decoding of content and production. Information was averaged for both orders and delays. Asterisks indicate significant information (n = 24, *P* < 0.05 corrected; *t* test, one tailed).

Electromagnetic artifacts and other, usually visually driven confounders often pollute the data in speech studies (35). Our source-level analysis provided evidence that content and production information were indeed speech related, originating from well-known speech-associated areas. In addition, we implemented a control analysis to test whether a possible visual cue confound had an impact on our results. Because the order of trial blocks was reversed in each participant’s second recording session, it was possible to cancel out the visual cue effect by decoding across sessions. To this end, we trained the cvMANOVA on one session and tested it on the other (Fig. 8).

Decoding across sessions was expected to be more challenging than decoding within a session, as the signal-to-noise ratio was impacted by additional variability due to head movement between the sessions. Nevertheless, we found significant information about both content and production in all relevant delays (Fig. 8). There was significant content information in both delays when it was instructed first (p_del1_ = 0.004, p_del2_ = 5.1 × 10^−5^; corrected) and in delay 2 when it was instructed second (*P* = 0.006; corrected). Production information was significant in delay 2 when content was instructed first (*P* = 3.4 × 10^−8^; corrected) and in both delays when production was instructed first (p_del1_ = 4.8 × 10^−5^, p_del2_ = 3.4 × 10^−6^; corrected). Thus, content and production information were stable across recording sessions and therefore also not driven by a visual, cue-related confound due to the sequential order of rule blocks within each session.

## Discussion

Our results shed light on the neural mechanisms underlying the flexible mapping between the content and motor production of human speech. Combining MEG, MVPA, and a factorial task design allowed us to dissociate content and production in the pre-execution phase, where key processing stages take place (7, 36–38) and electromagnetic artifacts of the motor production itself are ruled out (35).

Significant content information directly after the first cue suggests that content can be represented independently of a specific motor plan, which falls in line with implications from previous studies on the lexical and the sublexical syllable level (11–17, 19–24, 30). Therefore, the actual motor production is not necessary to form a neural representation of content even on the phoneme level. However, this does not imply that the content representation is completely independent of motor planning.

Content information was higher when vocalized, and production information was higher for the vowel /u/, implying a dependence of information strength on the degree of motor involvement. As previous studies have shown, overt and covert speech differ in terms of executive motor control, including M1 recruitment for the efference copy (15, 18, 23), which could account for the higher content information in vocalized trials. In addition, the stronger phonological code retrieval and encoding in overt speech could also contribute to the observed difference of content information between the production types (18). The two vowels also differ in motor involvement, as /ə/ is a nonarticulated innate-like vowel, whereas /u/ is strongly articulated and learned (39). Therefore, the stronger motor involvement could account for higher production information in the vowel /u/.

We located representations of content and production in the frontal and central cortex consistent with well-known speech-associated areas (2–7). The extension to temporal cortices may be due to efference copies in sensory regions (20–23, 29). Although one may not expect the higher-order language network to be recruited in our paradigm (33), we found neural representations of both content and production to be stronger in the left hemisphere. This could either indicate that language capacities beyond low-level speech were recruited or that low-level speech processes can already be lateralized under specific circumstances. Independently of these alternatives, our finding of lateralization in an early pre-execution phase suggests that the uncovered neural representations were indeed speech or language specific and did not reflect general working memory processes.

Our cross-decoding approach provided insights into the temporal dynamics and similarities between the neural representations of vocalization components. Our results suggest an overlap of neural representations of content and production during the first delay, when information about only one of the components was available to the subjects. One possible explanation for such an overlap could be an effort effect where conditions with a higher degree of motor involvement elicit higher neural activity than those with a lesser degree. Concretely, |u vs. ə| and |vocalized vs. imagined| could both correspond to contrasts of |high effort vs. low effort|. This effect could reflect priming motor signals preceding vocalizations (7, 40) and falls in line with studies finding stronger motor-related activations in overt than covert speech (15, 18). Alternatively, it could also reflect the firing patterns of one or more speech-specific neural populations that encode several content- and production-related features. Here, effort could drive either the firing rates of individual neurons or the number of recruited cells. Further invasive research is required to determine whether the same population or spatially close and therefore indistinguishable neurons are modulated by both content- and production-related effort.

In the second delay, cross-information between content and production was similar as in the first delay. However, as both content and production information were much higher, the representations were now clearly distinguishable. Moreover, cross-temporal decoding between both delays revealed that the content representation remained stable over time, whereas the production representation transformed once the content was known. Consequently, the divergence of the representations in the second delay was likely driven by the transformation of production. Taken together, this implies that the production representation during the second delay was a combination of the initial format during the first delay and an additional component. This additional component, building up once the content was known, may reflect the specific motor program used to prepare the articulation of the respective vowel, which complies with a description of distinct phoneme representations in the SMA (28) and syllable representations in the SMA, PMC, and M1 (2) in overt speech. In sum, our results show that, when isolated, both representations overlap and correlate with the degree of motor involvement. This overlap of the representations could be caused by premotor-like activity that is modulated by effort but independent of the actual execution. While the content representation remains stable, the production representation changes once the content is known, likely reflecting the addition of a specific motor program.

While natural speech can functionally be decomposed into content and motor production, there is little evidence for a content dimension on the neural level and even less is known about its dynamic interplay with motor production. Most implications for a neural content dimension come from studies focusing on the lexical or the sublexical syllable level (11–17, 19–24). Temporal dynamics have so far only been studied on the lexical level (36–38). Yet, the elementary building blocks of speech are phonemes, and to our knowledge, all previous research on this level is related to motor production (25–29). Our results uncover a neural content dimension for phonemes that was present independently of motor production and could therefore allow for a generalization between production forms. These results accord well with and expand the larger body of work on the higher sublexical and lexical levels.

Our findings set the stage for future research to investigate how neural codes of isolated phonemes and their motor production translate to those embedded in speech. For this, our combined approach of MEG, MVPA, and a rule-based paradigm provides a fruitful framework, thus opening a window for noninvasive speech research in health and disease.

## Methods

### Subjects.

Twenty-four healthy humans with normal or corrected-to-normal vision participated in the study (14 male; 21 right handed; mean age: 29 y; 5 y SD). All participants gave written informed consent before participation and received a monetary reward afterward. The study was conducted in accordance with the Declaration of Helsinki and approved by the ethics committee of the University of Tübingen.

### Behavioral Task and Stimuli.

Participants performed a rule-based vocalization task. In each trial, one of two vowels (/u/ or /ə/) had to be either overtly or covertly vocalized. Vowel and production type were instructed sequentially by visual cues. The corresponding rule, i.e., the assignment of the visual cues to their instructed content, changed across recording blocks and was indicated before the beginning of each block.

Participants self-paced the trials using closed-loop eye movement control. Each trial started with an initiation phase of 1,000 ms, during which a white fixation spot (diameter: 0.1° of the visual angle) appeared at the center of the screen. Once fixation was acquired, the first visual cue (a forward or backward white slash, length: 2°, width: 0.25° of the visual angle) appeared for 100 ms, instructing either content or production. Then, the fixation spot appeared again for a delay period of 2,000 ms. The second visual cue appeared for 100 ms, instructing the respective missing variable, followed by a second delay period displaying the fixation spot. Dimming the spot for 100 ms served as the go cue for the participants’ response. After the go cue, the fixation spot remained on screen for 1,500 ms, which gave time for the response of the participants. The intertrial interval was 1,000 ms long, indicated by dimming of the fixation spot. If fixation was broken after the onset of the first visual cue, the trial was aborted, which was indicated by a color change of the fixation spot to red for 500 ms. The cue configuration of the aborted trial was repeated at a random position later within this block.

Within each recording block, the order of instruction and the meaning of each instruction cue were fixed. However, in half of the blocks, the content was instructed first, whereas in the other half of the blocks, the production was instructed first. Moreover, the assignment of each visual cue to its meaning (content or production) was different in half of the blocks for each order. These four different rules led to four blocks of trials. Each block contained 80 trials, with 20 per condition (1: /u/ vocalized, 2: /u/ imagined, 3: /ə/ vocalized, and 4: /ə/ imagined). The order of the conditions was randomized per block. In total, there were 16 different conditions, including the different assignments of the visual cues to their instructed content. The 24 possible orders of the four blocks were randomly assigned to the participants.

Before the experiment, participants were instructed on how to articulate the vowels correctly. Thereby, we made sure that /u/ was clearly articulated, whereas /ə/ was produced with minimal involvement of the vocal tract. We also made sure that there was no involuntary articulatory movement visible for imagined vowels. During the experiment, the participants memorized the respective rule before each block. The rule was presented on the screen for as long as necessary. Eight training trials preceded each block to ensure correct performance. If necessary, the rule was shown again during the block while the sequence of trials was paused. All participants performed two MEG sessions with 320 trials each. For each participant, the order of blocks from the first session was inversed for the second session. During both sessions, the performance of the participants was monitored with a microphone and a camera. After the experiments, each trial was labeled for production type and in case of vocalized trials for vowels.

### Data Acquisition.

We recorded MEG (Omega 2000, CTF Systems Inc.) with 273 sensors at a sampling rate of 2,342.75 Hz in a magnetically shielded chamber. Participants sat upright with a screen at a 65-cm viewing distance. Stimuli were projected onto the screen by an LCD projector (Sanyo PLC-XP41, Moriguchi, Japan) with a refresh rate of 60 Hz. The projection was the only source of light in the chamber. Continuous head movement was monitored with three coils attached to fiducial points. In two participants, head movement could not be measured due to technical issues. Eye movements were recorded using an infrared eye tracker (EyeLink CL-OC, SR Research Ltd.) at a sampling rate of 1,000 Hz. For labeling of trials and vocal onset detection in vocalized trials, the participants’ responses were recorded with a microphone integrated into the MEG System, at a sampling rate of 2,343.75 Hz. An additional microphone (MD 419, Sennheiser electronic GmbH & Co KG) with a sampling rate of 44.1 kHz was used to record a higher-quality audio trace for the labeling of production types and vowels. In a separate session, we acquired structural T1-weighted MRIs (3 Tesla MAGNETOM, Siemens Healthcare GmbH) for source reconstruction based on each participant’s individual’s anatomy (resolution: 1 mm^3^, MPRAGE).

### Data Preprocessing.

Technically caused channel jumps were detected and corrected and time lags between digital triggers and actual stimulus presentation were corrected based on a photodiode signal. For one subject, we excluded a noisy channel from the analysis. We low-pass filtered the MEG data at 30 Hz (sixth order, zero-phase Butterworth infinite impulse response (IIR) filter), downsampled to 300 Hz and low-pass filtered the data, again, at 10 Hz. Each trial was baseline corrected using the 500 ms preceding the onset of the first visual cue. To reduce electromagnetic artifacts, we ran independent component analysis (ICA) on the data. To ensure convergence of the ICA algorithm, the data were high-pass filtered at 0.05 Hz. However, we applied the resulting unmixing matrix to the original data without any high-pass filter and removed artifact components like heartbeats and other small muscle activities. Vocal-onset detection in vocalized trials was possible for 37 sessions. Audio traces were missing due to technical issues in the remaining session. However, performance was closely monitored during the recordings and immediately corrected, if participants vocalized before the go cue appeared. The available audio traces were smoothed with a median-based sliding window model (window size 42.66 ms). A participant-wise threshold served for onset detection. For that, the rms of a 1,000 ms period of one of the imagined trials was calculated. The respective SD was multiplied by eight and added to the rms. In case of low vocalization amplitudes due to very soft voices of a few participants, the threshold was manually adjusted by slightly decreasing it until the onsets could reliably be detected. The vocalization was correctly performed, with an onset after the go cue, in 99% of the trials.

For all subsequent analyses, only correct trials were used. Those were trials with the correct production type and, in case of vocalized trials, with the correct vowel and a vocal onset after the go cue. For the nine sessions with a missing audio trace, only the correct vowel was considered.

### Cross-Validated MANOVA.

We estimated the amount of neural information about the variables of interest in the MEG data with cross-validated MANOVA (31, 32). As an extension of the commonly used cross-validated Mahalanobis distance, cvMANOVA allows for the simultaneous quantification of the variability in neural data due to several variables of interest. We performed 20 repetitions of cvMANOVA for each session with fivefold cross-validation. All folds and repetitions were subsequently averaged. We first estimated a noise covariance matrix using trials from all conditions. Next, we estimated contrasts of beta weights of each condition in a cross-validation fold’s training set, which accounted for the “training” of the model. The “testing” was done by estimating contrasts of beta weights in the respective fold’s test set. The dot product of these contrasts, normalized by the noise covariance, served as an estimate of the true pattern distinctness:

$$\begin{array}{c} D=\mathrm{trace}\left(\frac{1}{n}B_{\mathrm{train}}^{\prime }C_{\mathrm{train}}C_{\mathrm{train}}^{-1}X_{\mathrm{test}}^{\prime }X_{\mathrm{test}}C_{\mathrm{train}}C_{\mathrm{test}}^{-1}B_{\mathrm{test}}\Sigma^{-1}\right), \end{array}$$

where Σ^−1^ is the inverted noise covariance matrix, C_train_ is the contrast vector the model is trained on, C_test_ is the test contrast vector, and X_test_ is the design matrix indicating the unique condition of each trial in the test set. B_train_ and B_test_ contain the regression parameters of a multivariate general linear model:

$$B_{\mathrm{train}}=X_{\mathrm{train}}^{-1}Y_{\mathrm{train}},$$

$$B_{\mathrm{test}}=X_{\mathrm{test}}^{-1}Y_{\mathrm{test}},$$

where Y_train_ and Y_test_ are the training and test data sets. The inverted noise covariance matrix was estimated with the mean of the time window from cue 1 offset to go cue onset:

$$B_{{\mathrm{train}}_{\mathrm{tw}}}=X_{\mathrm{train}}^{-1}Y_{{\mathrm{train}}_{\mathrm{tw}}},$$

$$\Xi =Y_{{\mathrm{train}}_{\mathrm{tw}}}-X_{\mathrm{train}}B_{{\mathrm{train}}_{\mathrm{tw}}},$$

$$\Sigma^{-1}=\left(fE-p-1\right)\cdot {\left(\Xi^{'}\Xi \right)}^{-1},$$

with fE as the degrees of freedom and p as the number of sources.

Technically, cvMANOVA is a multivariate information-based cross-validated encoding approach. However, it shares many similarities with common multivariate decoding methods (41). The measure of neural information about the variables of interest can, theoretically, also be used to decode these variables on individual trials. Therefore, we refer to our results as decoding results.

For all sensor-level analyses, a subset of 137 approximately equally distributed sensors were included. This was to ensure a sufficient number of trials in relation to the degrees of freedom of the dataset.

### Cross-Decoding.

With cvMANOVA, we were able to decode across conditions by training and testing on different time points, variables, and levels of the variables. Therefore, the contrast vectors C_train_ and C_test_ were constructed to only contain the respective conditions to be trained or tested on. With this approach, we decoded across variables by using content (/u/ vs. /ə/) for the construction of the training contrast C_train_ and production type (vocalized vs. imagined) for the construction of the test contrast C_test_. To estimate content information for both production types separately, we constructed C_train_ based on trials including both production types, but C_test_ based on trials with only one production type, respectively. The same principle was applied for separately decoding the production type from both vowels. For decoding across time, we used the regression parameters B_train_ from one time point and B_test_ from another. We applied this to all pairs of time points. For cross-session decoding, we implemented a twofold cross-validation such that trials from session 1 and 2 served as training and test sets alternately.

### Expected Cross-Decoding.

To estimate a benchmark for the overlap between representations, we computed the expected cross-information (32). As the maximally possible amount of shared information between contexts depends on the information available in each individual context, the strength of the shared representation must be compared to the strength of both representations. Therefore, we estimated the expected cross-decoding

$$E_{12}=\sqrt{\left|D_{1}D_{2}\right|}\cdot \mathrm{sign}\left(D_{1}\right)\cdot \mathrm{sign}\left(D_{2}\right),$$

where D_1_ and D_2_ denote the pattern distinctness in the two contexts. If the representations were identical, the cross-decoding D_12_ would approach E_12_. Conclusively, cross-decoding values smaller than E_12_ indicate that the representations are not identical and, therefore, not fully overlapping.

### Source Estimation.

We generated individual single-shell head models (42) based on each subject’s structural T1-weighted MRI. Using linear spatial filtering (43), we estimated three-dimensional MEG source activity at 457 equally spaced locations ~7 mm beneath the skull. For searchlight analysis, we used the three dipole directions of each source and the respective immediate neighbors. The LI was computed by averaging the searchlight results for each hemisphere and subtracting right from left. Cortical areas were mapped according to the AAL atlas (44), and neural information was averaged for each area of the left hemisphere (*SI Appendix*, Fig. S4). For cross-session decoding, the three orientations were added, and a subset of 229 equally spaced sources was used for decoding.

### Statistical Analysis.

Neural information was averaged within two different time windows (delay 1: from 250 ms after cue 1 offset to cue 2 onset; delay 2: from 250 ms after cue 2 offset to go cue onset). Cross-temporal information within delay 1 was averaged over all conditions in which the relevant cue was instructed first, as information could only be present in these conditions. To estimate cross-temporal generalization between delays, we averaged an off-diagonal time window over those conditions where the relevant cue was presented first, as well as those mixed-order conditions where the relevant cue was presented first in one condition but second in the other. To estimate cross-temporal information within delay 2, we averaged data from all cue orders. For testing the significance of neural information and cross-information to be larger than 0, we employed one-tailed one-sample *t* tests. One-tailed paired *t* tests were applied for testing cross-time and cross-variable information to be smaller than the expected cross-information. For the comparison of content information in both production types and vice versa, we used two-tailed paired *t* tests. All *P*-values were false discovery rate (FDR) corrected for the number of tested time intervals (45).

### Potential Cue Confound.

We identified the source of a potential confound: due to the blocked design, in combination with possible nonstationarities in the dataset, content and production information could theoretically be influenced by representations of the physical cue itself, even though the cue appearance and its meaning were counterbalanced. Briefly, if noise led to independent shifts of the neural activity patterns of both cue options, this could mistakenly be identified as information about the variable indicated by the cue. To make sure that our results were not dependent on this potential confound, we took two measures. First, we excluded the 250 ms after cue onset in both time windows used for statistical analysis, as this was the time period that would likely be affected. Second, we performed cross-session decoding to confirm that content and production information were present if the possible cue confound was accounted for.

### Visualization.

For all line plots, data were smoothed with a 100-ms Hanning window (full width at half maximum).

### Software.

All analyses were performed using the Fieldtrip toolbox (46) and custom code in MATLAB.

## Supplementary Material

Appendix 01 (PDF)

## Data Availability

Preprocessed MEG data and analysis code to reproduce all reported results are publicly available at https://osf.io/5c43h/ (47).
