# Supplementary material for: Neural representations of the content and production of human vocalization
Source: Proc Natl Acad Sci U S A. 2023 May 30;120(23):e2219310120. doi: 10.1073/pnas.2219310120 (PMC10265962; doi:10.1073/pnas.2219310120)
Supplement: Supplementary file 1 — Appendix 01 (PDF) [file pnas.2219310120.sapp.pdf]

Supporting information

Neural representations of the content and production of human vocalization

Vera A. Voigtlaender[1,2,3,4,*], Florian Sandhaeger[1,2,3,4], David J. Hawellek[1,2,3,6], Steffen R. Hage[2,5] & Markus Siegel[1,2,3,*]

[1] Department of Neural Dynamics and Magnetoencephalography, Hertie Institute for Clinical Brain Research, University of Tübingen, Germany
[2] Centre for Integrative Neuroscience, University of Tübingen, Germany
[3] MEG Center, University of Tübingen, Germany
[4] Graduate Training Centre of Neuroscience, International Max Planck Research School, University of Tübingen, Germany
[5] Dept. of Otolaryngology-Head and Neck Surgery, Hearing Research Centre, University of Tübingen, Germany
[6] Roche, Pharmaceutical Research and Early Development, Roche Innovation Center Basel, Basel, Switzerland
*Correspondence: vera.voigtlaender@uni-tuebingen.de & markus.siegel@uni-tuebingen.de

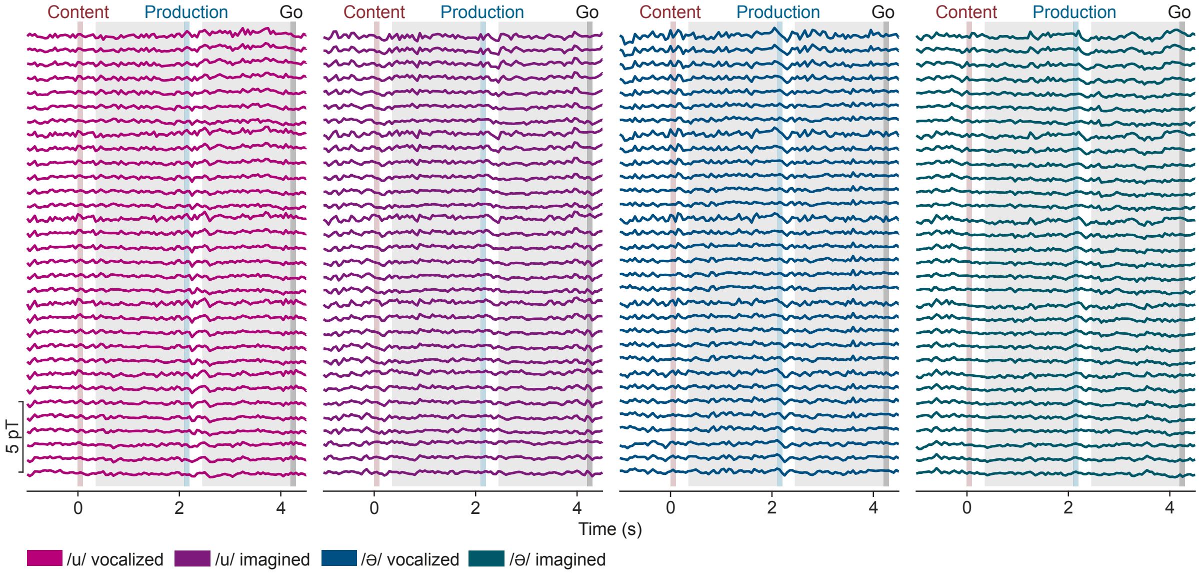

Fig. S1 | Preprocessed signals from left frontal sensors. Preprocessed single trial signals from 32 frontal sensors of the left hemisphere. Columns show four example trials from the order with content instructed first. Each trial has one of the four conditions: /u/ vocalized, /u/ imagined, /ə/ vocalized and /ə/ imagined.

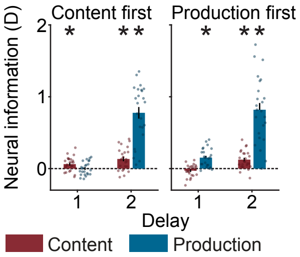

Fig. S2 | Neural information about content and production. Bar plots show average information in delay 1 and 2 with datapoints for individual subjects. Asterisks indicate significance (n = 24, $p < 0.05$ corrected; t-test, one-tailed).

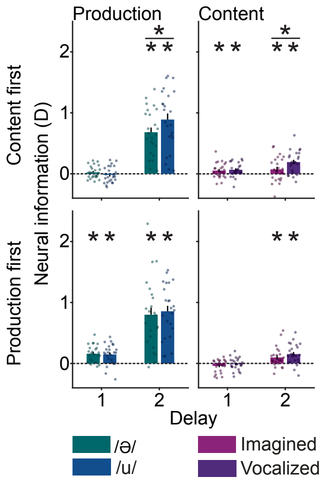

Fig. S3 | Neural information about content and production in split conditions. Bar plots show averaged information of delay 1 and 2 with datapoints for individual subjects. Asterisks above individual bars indicate significant information (n = 24, $p < 0.05$ corrected; t-test, one-tailed). Horizontal lines with asterisks on top indicate a significant difference (n = 24, $p < 0.05$ corrected; paired t-test, two-tailed).

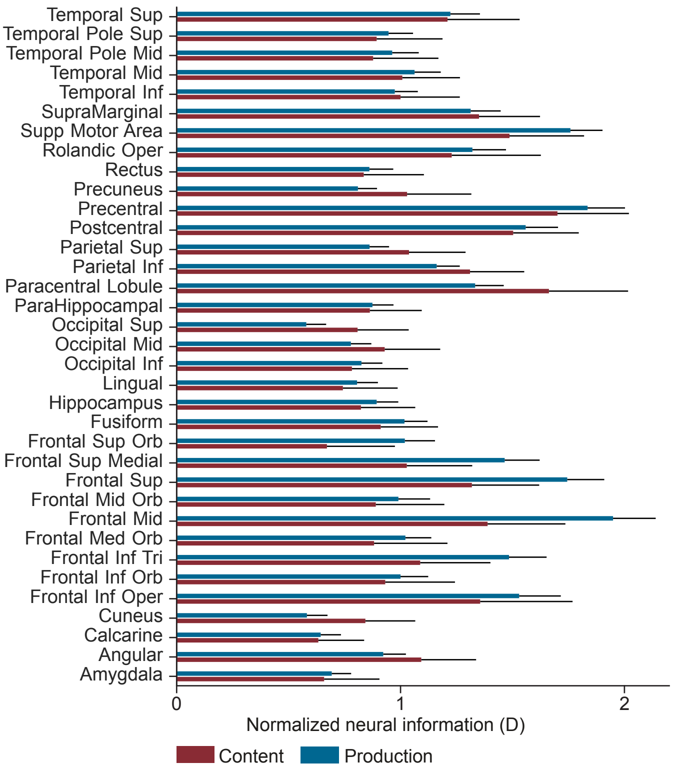

Fig. S4 | Spatial dynamics of neural information about content production. Production and content information in different areas of the left hemisphere (areas according to AAL atlas). Information was averaged over all delays with significant information, except for the 250 ms after the cues. The neural information about each variable was normalized by the respective median information across areas. Error bars indicate SEM. Significant information in all areas (n = 24, $p < 0.05$ corrected; t-test, one-tailed).

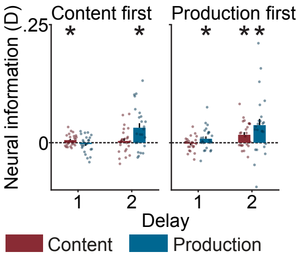

Fig. S5 | Lateralization index of content and production information. The lateralization index (left – right hemisphere information) was computed for both delays and both orders with datapoints for individual subjects. Asterisks indicate significance ($n = 24$, $p < 0.05$ corrected; t-test, one-tailed).
